# Predictors of nurses' work-related mental health during the COVID-19 pandemic: a paired follow-up study

**DOI:** 10.3389/frhs.2025.1583357

**Published:** 2025-04-25

**Authors:** Cicilia Nagel, Kerstin Nilsson

**Affiliations:** ^1^ Department of Laboratory Medicine, Lund University, Lund, Sweden; ^2^ Department of Health Sciences, Kristianstad University, Kristianstad, Sweden

**Keywords:** workplace, psychological phenomena, social support, registered nurses, health

## Abstract

Nurses are essential pillars of society, providing care for the sick. It is crucial for society to ensure that nurses have favorable working conditions, as they face a heightened risk of negative psychological conditions such as depression, anxiety, stress, and emotional exhaustion due to the demanding nature of their work. The strain on healthcare staff, particularly during the COVID-19 pandemic, has underscored these challenges. This study aimed to identify predictors of work-related mental health issues in nurses' work environments. In 2017, a questionnaire was distributed to 9,219 Swedish nurses, with 4,962 responses received. The questionnaire was redistributed during the pandemic in 2020, and 3,107 nurses responded. However, due to missing data and some nurses previously reporting a mental health diagnosis, only 2,030 questionnaires were included in the study. Among these, 143 nurses reported experiencing work-related mental health problems. The data was analyzed by logistic regression analysis, and 6 out of the 24 statements investigated were statistically significant (*p* < 0.05), i.e., there was a longitudinal association between nurses’ work situation and their mental health problems. The six statements were “I do not feel I get enough rest/recuperation between work shifts”, “The work pace in my daily work is too high”, “I do not feel I have enough support from my coworkers”, “I do not experience joy in my daily work”, “I do not feel like my daily work is meaningful”, and “I do not get enough opportunities at work to utilize my skills and knowledge”.

**Conclusions:**

This study revealed that work pace, recovery, support from colleagues, joy, meaningfulness, and development opportunities at work are particularly important for nurses' mental health. Actions in those areas are needed for nurses to have a sustainable work situation.

## 1 Introduction

During the COVID-19 pandemic, healthcare workers (HCWs), particularly nurses, were at the forefront, facing immense pressure. This situation significantly strained both nurses and healthcare organizations (1–5). Nurses constitute half of the global health workforce (6) and are often the initial point of contact for patients in healthcare facilities. The demanding nature of their work (7, 8) creates a heightened risk of developing mental health issues such as depression, anxiety, stress (9), and emotional exhaustion (10).

Depression, marked by persistent sadness and a loss of interest in activities, impacts various aspects of life, including relationships and job performance (11). Anxiety, although common, can escalate into disorders like generalized anxiety disorder (GAD) and panic disorder if persistent (12). Ignoring symptoms of depression and anxiety can exacerbate physical and emotional strain, negatively impacting patient care quality and increasing organizational workload (13, 14). Healthcare workers experience high levels of mental stress while caring for patients (15). The stress on nurses can result in serious consequences such as depression, reduced job satisfaction, and higher turnover rates (16). High professional stress levels are also associated with a diminished quality of life (17), physical health problems like migraines and muscle pain (18), and adverse effects on psychological well-being (19–21). Factors such as increased workloads, time pressure (16, 22, 23), lack of support (24), and the work environment (22, 23) contribute to negative stress, burnout symptoms (16, 23, 25), and job dissatisfaction (23). Emotional exhaustion refers to feelings of being emotionally overextended, drained of energy, and suffering from chronic fatigue (26). A systematic review published in 2024 (27) revealed that nurses are particularly susceptible to emotional exhaustion. Research indicates that long working hours, lack of control over work, limited participation in decision-making, poor social support, and insufficient support from nurse managers are linked to mental health issues (16, 20). Workload and work pressure affect job outcomes, leading to an increased risk of medical errors, decreased productivity (16), and compromised quality of care (28).

Studies show a high prevalence of mental health problems among nurses (29) and a rise in stress, anxiety, depression, and burnout symptoms among Swedish nurses (30). In Sweden and other countries, obtaining a work-related mental health diagnosis is challenging (31). Approved cases often relate to stress from high workloads or workplace bullying and harassment (29). While previous research has examined nurses' stress related to health and their work environment, comprehensive investigations into nurses’ overall work situations before and during the COVID-19 pandemic are limited. It is crucial to study the extraordinary work conditions during the pandemic as a natural stress test for healthcare organizations, affecting all areas related to nurses' self-reported work-related mental health diagnoses. Our previous research (30) identified differences in nurses' work-related mental health as well as their association with their work situation. This current study aims to further investigate predictors of work-related mental health problems in nurses' work situations through paired follow-up with nurses who developed work-related mental health problems between 2017 and fall/winter 2020.

## 2 Materials and methods

### 2.1 Surveys

This longitudinal study is part of the broader research initiative, “Sustainable Working Life for All Ages” (32, 33). The baseline survey, conducted in the spring of 2017, targeted all healthcare staff (*n* = 22,924) employed in the Region of Skane, the southernmost county in Sweden. An online questionnaire link was distributed via employees' work emails, resulting in 11,902 completed surveys (32). In 2017, 9,217 registered nurses (including specialist nurses) were employed in the region, with 4,692 (50.9%) completing the survey The follow-up survey took place from September to December 2020, involving all healthcare staff who were employed in 2017 and remained employed in 2020 (*n* = 18,143). The response rate for employed registered nurses in the region in 2020 was 40.1% (*n* = 3,107).

### 2.2 Study population

Out of the 3,107 replies, 477 had a previous mental health diagnosis, and 600 surveys had missing data, or the nurses had only answered the 2020 survey. Hence, the study population consisted of 2,030 registered nurses representing those who completed the paired follow-up survey. The study group consisted of nurses from the cohort who had no work-related mental health issues at baseline, were still employed in 2020, and had developed work-related mental health problems (*n* = 143). Examination of dropouts revealed that in both 2017 and 2020, 12 emails were undeliverable due to incorrect e-mail addresses. In spontaneous e-mail responses to researcher KN, employees cited various reasons for not completing the survey, including absenteeism, time constraints, and concerns about their managers discovering their responses. Unfortunately, it is not possible to accurately estimate the percentage of dropouts for these reasons.

### 2.3 Questionnaires

The original questionnaire, developed by researcher KN (32), included questions about respondents' sociodemographic characteristics, Karasek and Theorell's demand-control questions (34) and the nine impact and determinant areas for a healthy and sustainable working life as described in the theoretical SwAge model (35, 36) (see the Analysis Model paragraph for a brief description of the SwAge model). The 2017 questionnaire contained 158 questions, while the 2020 version included 41 additional COVID-19-specific questions created by researcher KN, infection control researchers, and virologists. The questionnaire was in Swedish. Some questions were yes/no, and others were open-ended, allowing participants to write freely. However, most questions in the current study used a Likert scale with four response options: fully agree (1), agree (2), disagree (3), and fully disagree (4). These options were later dichotomized into “agree” or “disagree”. Researcher KN collected and managed the sample data.

### 2.4 Themes in the analysis model

The theoretical SwAge model (Sustainable Working life for all Ages) (35, 36) was used as the theme areas in the analysis with the intention of investigating predictors of work-related mental health problems in nurses' work situations. The SwAge model consists of nine different impact and determinant areas that influence the ability and willingness to partake in working life, which relate to the four spheres of employability. Those four spheres and nine impact and determinant areas are as follows:

I. The health effects of work environments which include the following areas of determination:
  (1) The individual's diagnosis, self-rated health, and diverse functionality
  (2) The physical work environment with unilateral movements, heavy lifting, risk of accidents, climate, chemical exposure, and risk of contagion
  (3) Mental work environment, stress and fatigue syndrome, threats, and violence
  (4) Working hours, work pace, and possibility of recuperation during and between work shifts.
II. Financial incentives are associated with society's control of various financial carrots and sticks, such as through the social insurance system. Financial incentives include the following determinant areas:
  (5) The personal financial situation affects individuals' needs and willingness to work. Issues with employability due to ill health or lack of skills can cause individuals to be excluded from working life and have a poorer financial situation, e.g., through sick leave, unemployment, and early retirement, not least in tough times.
III. Relationships, social support, and participation, i.e., attitudes in the social context in which the individual finds himself/herself, whether the individual feels included or excluded from the group and receives sufficient social support from the environment when needed, include the following areas of determination:
  (6) The effects of the personal social environment, family, friends, and leisure contexts of and on work
  (7) The social work environment with leadership, norms at the workplace, group dynamics with colleagues, patients, etc., and the significance of the employment relationship context for individuals' work, participation and social support, bullying, victimization, and discrimination.
IV. The execution of work tasks and activities at work relates to individuals’ opportunity to perform their duties and inner satisfaction; this is also made possible by instrumental support and includes the following areas of determination:
  (8) Experience of motivation, appreciation, satisfaction and stimulation of work tasks and activities at work
  (9) The skills, knowledge, competence, and possibility for competence development for the individual's work activities and duties.

### 2.5 Variables

In the current study, eight out of the nine impact areas from the SwAge model were utilized in the analysis model. The impact area regarding personal finances (5) was primarily related to age-retirement possibilities and was therefore excluded from the current study's analysis. The first impact area (I), “The health effects of work environments” served as the outcome measure. Respondents were asked if they had a diagnosis of ill health or injury caused by their work (yes/no). If they answered yes, they were provided with options for diagnoses from the WHO's International Classification of Disease-10 (ICD-10) principal diagnosis codes to self-report their diagnosis. Diagnoses of depression, anxiety, emotional exhaustion, and stress symptoms were combined and analyzed as the dependent variable “mental health,” representing work-related mental health diagnoses.

The analysis included 24 statements about the nurses' work situation, categorized into seven of the nine impact areas of the SwAge model. The examined impact areas were: (2) physical work environment (two statements); (3) mental work environment (five statements); (4) work pace, work time, and recuperation (three statements); (6) personal social environment (two statements); (7) social work environment, organization, and leadership situation (six statements); (8) motivation and satisfaction with work tasks (four statements); and (9) knowledge and competence (two statements).

### 2.6 Statistical analyses

The participants' sociodemographic characteristics and the responses (in percentages) of the 2,030 participants, including those without a work-related mental health diagnosis and those with a diagnosis (*n* = 143), to the 24 statements were descriptively analyzed.

A logistic regression analysis was performed to generate simple estimates and build multiple regression models [odds ratios (ORs) with 95% confidence intervals (CIs)] to explore the associations between various factors and work-related mental health issues in 2020. A subgroup of 143 individuals, who had no mental health diagnosis in 2017 but reported one in 2020, were examined to assess longitudinal association between mental health diagnoses and variables from the seven determinant areas.

Initially, to investigate the associations of the impact areas with nurses' mental health, each of the 24 statements was analyzed with simple regression against the dependent variable (Table 1). Subsequently, the statements within each influence area were analyzed step by step to construct an influence area model. Statistically significant statements (*p* value <0.05 and CI not including 1.00) were included in a model for each influence area. In the next step, each eliminated statement was added one at a time to evaluate the robustness of the model for each influence area.

**Table 1:** Simple estimates and multiple regression model inside determinant areas of the SwAge model, i.e., the statements in the different areas in association with work-related mental health diagnoses among nurses who developed a mental health diagnosis between 2017 and 2020 (*n* = 143).

Area of the SwAge-model	Statement	Simple		Multiple	
		OR	95% CI	OR	95% CI
Physical work environment	My current work is too physically straining for my health	1.27	1.00–1.92	1.22	*
	For the most part I cannot cope with the physical work demands	2.12	1.18–3.83	2.14	1.18–3.91
Mental work environment	My work involves many mentally heavy work tasks	1.81	1.24–2.64	1.33	*
	I wish for more opportunities to determine how to perform my work	1.53	1.09–2.16	1.02	*
	I wish for greater control over my work	1.65	1.17–2.52	1.19	*
	At my work there are not enough possibilities to be reallocated to less demanding work tasks for those who need it	1.12	*	1.18	*
	My work tasks usually clump together to that extent that I get frustrated	2.23	1.56–3.23	2.14	1.46–3.07
	Not having enough staff means that I cannot perform my work	1.73	1.22–2.45	1.23	*
Work pace, work time, recuperation	I do not feel like I get enough rest/recuperation between work shifts	1.87	1.32–2.63	1.72	1.19–2.49
	I do not have time to perform the work duties I have planned for the day	1.88	1.32–2.68	1.45	*
	The work pace in my daily work is too high	1.96	1.39–2.76	1.51	*
Private social environment	I want to spend more time at leisure activities and will therefore work less in the future	1.12	*	1.08	*
	I need to work more at home/care for relatives, and will therefore work less in the future	1.14	*	1.15	*
Social work environment	The social community at my workplace does not make me want to stay	1.82	1.24–2.67	1.50	*
	Big changes in my work situation cause me to want to leave	1.19	*	1.09	*
	I do not feel I have enough support from my closest manager	1.11	*	1.18	*
	I do not feel I have enough support from my coworkers	2.55	1.62–4.03	2.43	1.46–4.05
	I feel bullied or shut out from the community at my workplace	1.46	*	1.16	*
Motivation and satisfaction of and to work tasks	I do not feel like my daily work is meaningful	1.20	*	1.74	*
	I do not feel like my work is stimulating	1.30	*	1.30	*
	I do not experience joy in my daily work	2.83	1.94–4.14	3.28	1.78–6.04
	I do not experience satisfaction in my daily work	2.13	1.45–3.13	1.13	*
Knowledge and Competency	I do not get enough opportunities at work to utilize my skills and knowledge	2.49	1.66–3.75	1.71	1.05–2.89
	I do not feel like my competencies are being utilized in a satisfactory way	1.87	1.23–2.76	1.18	*

* Not statistically significant. OR, odds ratio; CI, confidence interval. *P* < 0.05.

Finally, a multiple regression model was developed to examine the effects of all influence areas on nurses' mental health. Statistically significant statements from each dependent area in the initial step were examined in a multiple regression model, with each influence area added step by step. Statements that remained statistically significant (*p* value <0.05 and CI not including 1.00) were included in the model, and each eliminated statement (*p* value >0.05 and CI including 1.00) was added one at a time to test the robustness of the total multiple model of all influence areas' effects on nurses' mental health (Table 2). The analysis was conducted using IBM SPSS software.

**Table 2:** The final joint multiple regression model. Factors significantly related to work-related mental health diagnoses among nurses who developed a mental health diagnosis between 2017 and 2020 (*n* = 143).

Area of the SwAge-model	Statement	OR	95% CI
Work time, work pace, recuperation	I do not feel I get enough rest/recuperation between work shifts	1.40	1.04–2.05
	The work pace in my daily work is too high	1.62	1.11–2.37
Social work environment	I do not feel I have enough support from my coworkers	1.57	1.05–2.90
Motivation and satisfaction of and to work tasks	I do not experience joy in my daily work	2.34	1.45–3.78
	I do not feel like my daily work is meaningful	2.49	1.12–5.54
Knowledge and Competency	I do not get enough opportunities at work to utilize my skills and knowledge	2.33	1.44–3.76

OR, odds ratio; CI, confidence interval; Nagelkerke *R*^2^ = 0.066. Tolerance = >0.8. Hosmer-Lemeshov 0.981. *P* < 0.05.

### 2.7 Ethical considerations

The study was conducted in compliance with Swedish law (37) and the Helsinki Declaration (38). The potential benefits of the knowledge generated by this study are deemed to outweigh any potential risks. Data handling and storage adhered to university policies and GDPR guidelines for managing sensitive information (39). The study received approval from the Swedish Ethical Review Agency (approval numbers 2016/867 and 2020-01897).

## 3 Results

### 3.1 Participants' sociodemographic characteristics

Among the 143 participants who reported a mental health diagnosis in 2020, the median age was 50, ranging from 28 to 68 years. Women comprised 92.3% of the respondents. Additionally, a majority (52.4%) of the participants had over 16 years of experience working as nurses (Table 3).

**Table 3:** Sociodemographic characteristics of the respondents in the 2020 survey compared to those who did not have a self-reported work-related mental health diagnosis.

Characteristic	Mental health diagnoses 2020 *n* = 143	No mental health diagnoses 2020 *n* = 1,887
Age md (min–max)	50 (28–68)	53 (26–70)
Female gender (%)	92.3	90.1
Country of birth:		
Sweden (%)	85.3	89.1
Nordic countries (%)	5.6	2.2
Europe (%)	6.3	5.2
Outside of Europe (%)	2.8	3.5
Civil status:		
Married/live-in partner (%)	75.5	77.5
Living apart together (%)	3.5	4.7
Single (%)	21.0	17.8
Children living at home:		
No children living at home (%)	41.3	43.6
0–3 years of age (%)	13.3	9.2
4–6 years of age (%)	13.3	11.2
7–12 years of age (%)	25.2	19.9
13–15 years of age (%)	19.6	12.6
16–19 years of age (%)	13.3	16.9
Years in the profession:		
<5 (%)	13.3	11.6
6–10 (%)	18.9	13.2
11–15 (%)	15.4	14.6
>16 (%)	52.4	60.6

### 3.2 Findings

#### 3.2.1 The nurses' experience of their work situation

The analyzed questionnaires had no missing data. A descriptive analysis comparing those without a work-related mental health diagnosis to those who developed such a diagnosis in 2020 revealed differences in their work situation experiences (Table 4). The statements with highest OR were found in the following areas: *Physical work environment, Mental work environment, Work pace, work time, and recuperation,* S*ocial work environment* and *Motivation and satisfaction of and to work tasks.*

**Table 4:** A descriptive table of all the 2,030 participants who responded (in percentages) to the 24 statements included in the study.

Area of the SwAge-model	Statement	Mental health diagnoses 2020 (*n* = 143)	No mental health diagnoses 2020 (*n* = 1,887)
		Agree	Agree
Physical work environment	My current work is too physically straining for my health	21.7	18.3
	For the most part, I cannot cope with the physical work demands	11.9	4.9
Mental work environment	My work involves many psychologically heavy work tasks	71.7	58.9
	I wish for more opportunities to determine how to perform my work	53.8	43.6
	I wish for greater control over my work	51.4	39.1
	At my work there are not enough possibilities to be reallocated to less demanding work tasks for those who need it	58.7	60.9
	My work tasks usually clump together to the extent that I get frustrated	60.1	39.5
	Not having enough staff means that I cannot perform my work	60.1	47.5
Work pace, work time, recuperation	I do not feel like I get enough rest/recuperation between work shifts	55.9	40.6
	I do not have time to perform the work duties I have planned for the day	41.3	27.1
	The work pace in my daily work is too high	57.3	40.4
Private social environment	I want to spend more time at leisure activities and will therefore work less in the future	79.4	77.9
	I need to work more at home/care for relatives, and will therefore work less in the future	23.6	21.4
Social work environment	The social community at my workplace does not make me want to stay	28.8	18.7
	Big changes in my work situation cause me to want to leave	19.6	16.7
	I do not feel I have enough support from my closest manager	34.3	31.8
	I do not feel I have enough support from my coworkers	18.9	8.9
	I feel bullied or shut out from the community at my workplace	4.2	2.9
Motivation and satisfaction of and to work tasks	I do not feel like my daily work is meaningful	6.9	5.4
	I do not feel like my work is stimulating	13.7	9.9
	I do not experience joy in my daily work	30.8	13.6
	I do not experience satisfaction in my daily work	28.0	15.4
Knowledge and Competency	I do not get enough opportunities at work to utilize my skills and knowledge	24.9	11.8
	I do not feel like my competencies are being utilized in a satisfactory way	27.3	17.1

#### 3.2.2 Simple estimates and multiple regression models relations to work-related mental health

According to the simple estimates, the six statements that showed the highest OR were “I do not experience joy in my daily work” (OR 2.83, CI 1.94–4.14); “I do not feel that I have enough support from my coworkers” (OR 2.55, CI 1.62–4.03); “I do not have enough opportunities at work to utilize my skills and knowledge” (OR 2.49, CI 1.66–3.75); “My work tasks usually clump together to the extent that I get frustrated” (OR 2.23, CI 1.56–3.23); “I do not experience satisfaction in my daily work” (OR 2.13, CI 1.45–3.13); and “For the most part, I cannot cope with the physical work demands” (OR 2.12, CI 1.18–3.83).

For the multiple variables, we found that the statements that showed the highest OR were “I do not experience joy in my daily work” (OR 3.28, CI 1.78–6.04), “I do not feel I have enough support from my coworkers” (OR 2.43, CI 1.46–4.05), “For the most part, I cannot cope with physical work demands” (OR 2.14, CI 1.18–3.91) and “My work tasks usually clump together to the extent that I get frustrated” (OR 2.14, CI 1.46–3.07) (Table 1).

#### 3.2.3 Final multiple regression model

In the final step of the analysis, a comprehensive multiple regression model was developed. Statistically significant variables were included in a joint model encompassing all examined areas of the SwAge model. This model was assessed for robustness and stability by sequentially adding each excluded variable until the final multiple regression model was established. The final model identified the three strongest predictors of nurses' work-related mental health diagnoses as: “I do not feel like my daily work is meaningful” (OR 2.49, CI 1.12–5.54), “I do not experience joy in my daily work” (OR 2.34, CI 1.45–3.78), and “I do not get enough opportunities at work to utilize my skills and knowledge” (OR 2.33, CI 1.44–3.76) (Table 2).

The Nagelkerke *R*^2^ value indicated that the final joint multiple regression model could explain a 6.6% proportion of variance. The Hosmer-Lemeshow test supports the model's validity, which yielded a score greater than 0.05. This result implies that the model's estimates fit the observed data well, indicating an acceptable level of goodness-of-fit. Additionally, the tolerance values for the variables in the model were all greater than 0.40. This indicates a low risk of multicollinearity, meaning that the independent variables in the model are not highly correlated with each other. This enhances the reliability and stability of the model's estimates. Overall, the final joint multiple regression model can be considered as both meaningful and robust, thus providing valuable insights into the factors associated with work-related mental health diagnoses among the participants.

## 4 Discussion

Nurses are considered one of society's mainstays; however, many in the profession suffer from work-related mental illness. The current study aimed to identify the areas and factors most critical for nurses developing work-related mental illness over time, based on the SwAge model's comprehensive approach to a sustainable working life (34, 35). We used a cohort of nurses who were without mental illness in 2017. However, possibly due to the extreme strain of the COVID-19 pandemic on their work situation, some nurses developed work-related mental health diagnoses by 2020. The results showed that nurses who did not find their daily work meaningful and joyful, lacked sufficient rest and recuperation between shifts, had limited opportunities to utilize their skills and knowledge, felt the work pace was too high, and did not receive enough support from coworkers were at increased odds of developing work-related mental health issues. Therefore, addressing these areas is crucial to providing nurses with a more sustainable working life. The final joint multiple regression model identified the most important areas for the development of work-related mental illness as: motivation and satisfaction with work tasks, knowledge and competence, work time, work pace, recuperation, and the social work environment.

### 4.1 Motivation and satisfaction of and to work tasks

In the current study, the strongest associations with work-related mental health issues were not finding work tasks meaningful, followed by not experiencing joy in daily work. Previous research has emphasized the importance of performing core professional tasks and deriving satisfaction from doing a good job and receiving recognition for it to find work meaningful (33, 40, 41). When employees are overwhelmed with too many tasks, tasks outside their professional role, or receive no internal or external rewards, they perceive their work as unsatisfying, less stimulating, or pointless (40). A previous study found a positive relationship between meaningful work and job embeddedness, with job embeddedness mediating the effect of meaningful work on turnover intention (42). Despite the high turnover rate among nurses globally, the sense of meaningful work may be why many nurses remain in healthcare (43). Additionally, experiencing joy at work is crucial for coping with the stressors of the nursing profession. Joy can serve as a coping strategy, especially during demanding times like the COVID-19 pandemic. Research has shown that meeting healthcare workers' needs for joy at work is essential (44). Appreciation from others fosters joy at work and contributes to a positive work climate with camaraderie and teamwork (45). Health care leaders need to understand the factors that diminish joy at work to improve working conditions for healthcare professionals (46).

### 4.2 Knowledge and competency

The results of the current study highlighted the importance of knowledge and competence for nurses' work-related mental well-being. Previous research has shown that having the appropriate knowledge to perform tasks is crucial for delivering quality work, feeling calm and secure in one's role, and managing work-related stress (14, 33, 35). This was especially true during the high-pressure situation of the COVID-19 pandemic before the virus's transmission routes were fully understood (20). The current study found a slightly increased odds of work-related mental illness among younger nurses with less experience, although this difference has been more pronounced in other studies (20). In professions involving direct contact and care, such as nursing, experiential knowledge—gained over a lifetime and through work experience—is vital for effectively treating and interacting with patients (14, 33, 35, 40). Additionally, the current study found a significant correlation between work-related mental health diagnoses and the lack of opportunities to utilize one's skills and knowledge. When nurses feel their expertise is underutilized, it can lead to decreased motivation, prompting them to seek other employment or develop work-related mental health issues. Ongoing staff development is also seen as beneficial for adapting to the rapid changes in healthcare (47).

### 4.3 Social work environment

The current study found that nurses lacked support from their co-workers. Previous studies highlight the significant impact of the social work environment, including relationships with coworkers and leadership, on nurses’ work-related psychological well-being (20, 48) and job performance (48). Lacking sufficient support from coworkers can negatively affect their well-being. During stressful situations, such as the initial stage of the COVID-19 pandemic, organizational and leadership concerns about work organization can disrupt work groups, increasing the risk of scapegoating and reducing social support (20). Work groups that manage uncertainty well and where employees support and trust each other tend to handle work situations and tasks better than those lacking cohesion and social support. Social support is multifaceted and influenced by various factors (14, 33, 35, 40, 49). Support from co-workers has been shown to enhance job satisfaction (25, 30, 32, 50), improve the quality of care, help nurses manage stressful work situations (40, 45), and enable them to provide effective and holistic patient care (51). The British Psychological Society (BPS) issued guidelines to support the mental well-being of healthcare workers during the pandemic, emphasizing the importance of continued peer support and normalizing feelings of anxiety (52).

### 4.4 Work time, work pace, and recuperation

The final multiple regression model in the current study indicated that insufficient rest between work shifts and high work pace were significant predictors of developing work-related mental health issues. A high work pace was correlated with mental health diagnoses in the current study. The ability to recover both during and between work shifts is crucial for managing work demands and maintaining individual health (14, 33, 35). A recent study found that rest breaks are effective in decreasing professional burnout among registered nurses (53). Previous research (30) found that inadequate rest contributes to poor mental health. Studies have shown that staff shortages lead to higher workloads, which negatively impact on job satisfaction (54). A study from Lebanon revealed that nurses with heavier workloads and poorer teamwork climates had higher odds of developing mental health conditions, affecting various aspects of their health, and increasing the risk of comorbidities (55). Balancing work and leisure, as well as ensuring sufficient recovery, is challenging without a manageable workload.

The current study found an association between lack of recuperation during work shifts and the development of work-related mental health diagnoses. A Finnish study showed that nurses working at a higher physical intensity, under increased time pressure, and experiencing mental strain had reduced recovery from work (56). Sleep is a fundamental human need, essential for proper functioning (57). Participants in a previous study reported difficulty falling asleep, attributing it to insufficient time to mentally decompress despite physical exhaustion (58). Lack of rest or recuperation can lead to decreased concentration and other somatic problems (59). Demanding work schedules can hinder recuperation between shifts, contributing to fatigue, increased risk of work-related injuries, and burnout (60). Reporting work-related injuries or illnesses is often a lengthy process and rarely approved, as it is challenging to prove they are solely caused by work (31). Consequently, many individuals refrain from reporting their injuries or illnesses. However, many believe their injuries or illnesses would not have occurred if not for their work situation. The COVID-19 pandemic has underscored the necessity for nurses to be in good physical and mental health to provide quality patient care (61).

### 4.5 Limitations and strengths

One limitation of this study is the substantial percentage of non-responders, with response rates of 50.9% in 2017 and 40.1% in 2020. However, given the nature of surveys, a low response rate was anticipated. To try to reduce non-response bias the surveys were open for two months during which time follow-up e-mails were sent out to encourage participation. To mitigate sample selection bias, we tried to ensure diverse representation by inviting all registered nurses employed in the Region to partake in the study. There is a risk of a healthy worker effect, as individuals with more pronounced depression may be on sick leave or unable to complete the questionnaire. We attempted to mitigate this by including a broad exposure group. It is always possible to lose participants during research if they become too ill to continue working. A strength is that all nurses employed in the Region of Skane in 2017 and 2020 and who did not have a prior diagnosis of stress, burnout, depression, or anxiety were considered viable participants. Nurses who completed the survey but had a previous mental health diagnosis, as well as those who completed only one of the surveys, were excluded as were questionnaires with missing data. Regarding dropout analysis, we can only track those currently employed in the participating healthcare organizations in southern Sweden. We can only speculate whether nurses who left their employment did so due to changing workplaces, leaving the profession, retiring, or passing away. We do not know if non-responders were on parental leave, sick leave, unwilling to participate, or simply lacked time. Another limitation is that the analysis included only 24 statements, with an unequal number of statements across the seven examined areas of the SwAge model, which could influence the results. However, addressing seven areas is more comprehensive than most studies on nurses' complex work situations, as identified through a systematic review of published studies in international peer-reviewed journals (20). We chose logistic regression analysis for this study and consider it a satisfactory method. However, alternative methods, such as factor analysis, could compute indexes for the dependent areas. As Sloan et al. (62) suggested, single-item measurements are less reliable than indexes. Conversely, Matthews et al. (63) argued that using single-item measures does not indicate a weak research design and that it is possible to develop measures that accurately and reliably represent a given construct. A strength of this study is the diverse representation of nurses from various work areas, tasks, and specialties. The study group is representative for the study population. This broad representation enhances the generalizability of the findings to registered nurses in general who are employed in a similar setting. One limitation is that the results only include those nurses that decided to stay (or come back) in the Region between 2017 and 2020, and therefore the predictors for nurses who left their employment could vary. Another strength is that participants had four response options for the statements, ensuring all data were utilized.

## 5 Conclusions

The results of this study suggest a longitudinal association between nurses’ work-related mental health diagnoses and several factors: lack of joy and meaningfulness in their work, insufficient skills and knowledge, inadequate support from coworkers, insufficient recuperation between shifts, and a high work pace. Addressing these areas is crucial to providing nurses with a more sustainable mental working life. In recent years, nurses have faced high-pressure work environments, a situation exacerbated by the COVID-19 pandemic. Unfortunately, the outlook remains challenging, with a growing shortage of healthcare professionals expected to persist. The findings from this study can guide hospitals, health ministries, and other relevant organizations in taking action to improve nurses' working conditions and quality of life at work, as well as in developing interventions that effectively address their current mental health needs.

## Data Availability

The raw data supporting the conclusions of this article will be made available by the authors, without undue reservation.
